# Von Willebrand Factor Inhibits Mature Smooth Muscle Gene Expression through Impairment of Notch Signaling

**DOI:** 10.1371/journal.pone.0075808

**Published:** 2013-09-23

**Authors:** He Meng, Xiaojie Zhang, Soo Jung Lee, Michael M. Wang

**Affiliations:** 1 Department of Neurology, University of Michigan, Ann Arbor, Michigan, United States of America; 2 Department Molecular and Integrative Physiology, University of Michigan, Ann Arbor, Michigan, United States of America; 3 Neurology Service, VA Ann Arbor Healthcare System, Ann Arbor, Michigan, United States of America; INSERM, France

## Abstract

Von Willebrand factor (vWF), a hemostatic protein normally synthesized and stored by endothelial cells and platelets, has been localized beyond the endothelium in vascular disease states. Previous studies have implicated potential non-hemostatic functions of vWF, but signaling mechanisms underlying its effects are currently undefined. We present evidence that vWF breaches the endothelium and is expressed in a transmural distribution pattern in cerebral small vessel disease (SVD). To determine the potential molecular consequences of vWF permeation into the vessel wall, we also tested whether vWF impairs Notch regulation of key smooth muscle marker genes. In a co-culture system using Notch ligand expressing cells to stimulate Notch in A7R5 cells, vWF strongly inhibited both the Notch pathway and the activation of mature smooth muscle gene promoters. Similar repressive effects were observed in primary human cerebral vascular smooth muscle cells. Expression of the intracellular domain of NOTCH3 allowed cells to bypass the inhibitory effects of vWF. Moreover, vWF forms molecular complexes with all four mammalian Notch ectodomains, suggesting a novel function of vWF as an extracellular inhibitor of Notch signaling. In sum, these studies demonstrate vWF in the vessel wall as a common feature of cerebral SVD; furthermore, we provide a plausible mechanism by which non-hemostatic vWF may play a novel role in the promotion of vascular disease.

## Introduction

Small vessel disease (SVD) of the brain is a common cause of stroke and vascular dementia and plays an important role as a cofactor for Alzheimer's dementia and Parkinson's disease [1], [2], [3], [4], [5], [6], [7], [8], [9]. Despite extensive characterization of the histopathology of SVD over a half century ago, the molecular pathways that drive vessel degeneration remain incompletely understood.

Vascular smooth muscle cell disease has emerged as a key feature of SVD. Pathological evidence of sporadic SVD reveals medial hypertrophy followed by loss of smooth muscle cells combined with fibrosis of the vascular media [10], [11]. In addition, genetic analysis has clearly implicated smooth muscle disease in familial SVD. For example, cerebral autosomal dominant arteriopathy with subcortical infarcts and leukoencephalopathy (CADASIL), the most common inherited cause of vascular dementia [12], is caused by mutations in NOTCH3 [13], a gene preferentially expressed in vascular smooth muscle [14], [15]. Furthermore, mutations in COL4A1, a gene encoding the main component of smooth muscle basement membranes, also causes SVD in families with inherited leukoencephalopathy [16], [17], [18]. Pathways by which mutant NOTCH3 and COL4A1 lead to smooth muscle dysfunction and SVD are under active investigation.

A number of cellular and molecular changes have been accepted as markers of vascular smooth muscle pathology. In healthy vessels, smooth muscle cells are non-mitotic and exhibit high expression of mature smooth muscle genes, including smooth muscle alpha-actin (SMA) [19], [20], smooth muscle myosin heavy chain (SM-MHC) [21], [22], SM22-alpha [23], and h-calponin [23]. In contrast, in disease states, smooth muscle cells proliferate and repress expression of these genes.

The large hemostatic protein von Willebrand factor (vWF) accumulates in both small and large vessels of the brain in CADASIL [24]. Of note, vWF binds to smooth muscle cells and rapidly alters expression of the aforementioned vascular smooth muscle genes. The presence of vWF in the vessel wall and the biological activity of the protein in cell signaling assays suggest that vWF could participate in vascular pathology via a novel molecular function that is independent of its canonical role in hemostasis.

In this study, we show that transmural arterial vWF deposition occurs in a multitude of disorders that feature cerebral small vessel pathology. The putative receptor-mediated pathways underlying these novel cellular actions of vWF are unknown. Given that Notch signaling upregulates critical genes in differentiated smooth muscle [25], [26], [27], [28], we also tested the hypothesis that vWF inhibits the expression of smooth muscle genes by attenuating Notch signaling.

## Materials and Methods

### Immunohistochemistry

Brain samples were obtained from the Michigan ADRC (grant P50-AG08671) and the NICHD Brain and Tissue Bank for Developmental Disorders at the University of Maryland, contract HHSN275200900011C, Ref. No. NO1-HD-9-0011. Formalin fixed, paraffin embedded brain tissue was analyzed using vWF antibodies as described before using a rabbit polyclonal antibody (Dako) in conjunction with the Dako autostaining system. Confirmatory staining was performed with the monoclonal F8/86 against vWF. vWF preprotein (vWF-pp) monoclonal 239.7 was used under the same conditions, and results were confirmed with 239.8; both vWF-pp antibodies produced an identical pattern in human brain. SMA staining was performed using the mouse monoclonal 1A4 (Santa Cruz). Double staining was performed by first applying using mouse antibody 239.7 that was detected with an alkaline phosphatase secondary antibody and a blue NBT/BCIP reaction product. The second step of the procedure used rabbit antibodies against mature vWF detected by an HRP secondary antibody and brown DAB staining. Double stained tissues were not counterstained.

### Cell culture and luciferase reporter assays

293A (Qbiogene), L fibroblasts (control parental “L cells” and Notch ligand producing cell lines stably transfected with Jagged1 or Delta1; [29]), and A7R5 (ATCC) were grown in Dulbecco's modified Eagle's medium (Invitrogen) supplemented with 10% fetal bovine serum (Invitrogen). H460 cells were propagated in RPMI 1640 with 10% fetal bovine serum (Invitrogen). Human brain vascular smooth muscle cells were purchased and grown in smooth muscle cell media from the same manufacturer (ScienCell Research Labs). For luciferase assays, Notch signal receiving subconfluent cells (H460 or A7R5) were first transfected with two constructs: 1) a cloned promoter fused to firefly-luciferase and 2) a Renilla luciferase reporter driven by the TK promoter which was used to normalize transfection efficiencies within each experiment [30], [31]. The cloned firefly luciferase promoter constructs, including Hes1, SMA, SM22, and MHC, have been previously described {Meng, 2012 #135}. After an overnight incubation, an equal number of ligand producing cells (L, Jagged1 or Delta1 cells) were overlayed on the Notch signal receiving cells. At the same time, purified recombinant vWF (Haematologic Technologies; Factor VIII-free) or vehicle was added directly to the culture medium. After an additional 24 hours, cell lysates were harvested and a dual luciferase assays was used to quantify expression of transfected promoters. The ratio of firefly luciferase to renilla luciferase reflected the activity of the promoter of interest.

Endogenous Notch-dependent gene expression was measured by quantitative RT-PCR. These experiments were performed in a similar manner as luciferase assays of Notch function, except Notch receptor expressing cells were not transfected. As in luciferase assays, vWF was added to the culture media after coculture with L cells expressing Notch ligands. Notch target gene expression was measured from RNA isolated from the co-culture. To assay expression of the target genes specifically in Notch receptor expressing cells, human or rat specific primers were used that did not cross react with murine nucleic acids derived from ligand producing cells.

### Western blots and coimmunoprecipitation

Myc (9E10, Santa Cruz), hemagglutinin (Santa Cruz) or V5 (Invitrogen) monoclonal antibodies have been previously described and were used at 10–50 ng/ml [30]. Jagged polyclonal (Santa Cruz) and NOTCH3 monoclonal (Abnova) antibodies were used for Western blotting. Immunoprecipitation was performed at 4°C as previously described [30], [31]. The cDNA construct for full length, C-terminally myc-(His)6-tagged human vWF in pCDNA3.1 was a gift from Dr. Karl Desch and David Ginsburg [32]. Tagged Notch1-4 ectodomains and Jagged1 clones in immunoprecipitation experiments have been previously described [33], [34] and contain human sequences cloned in frame with either V5 or HA tags at the C-terminus. Full length cDNA constructs for human NOTCH3 and CADASIL point mutants have also been described [33].

### Solid phase binding assays

The following purified recombinant proteins were obtained from R&D Systems: recombinant fragments of human Notch-1-Fc, Notch2-Fc, and Notch-3-Fc (first 11 EGF repeats), rat Jagged1-Fc, and control human IgG1 Fc. Purified human vWF (free of Factor VIII) was purchased from Haematological Technologies. Solid phase assays were performed as described in previous published work on NOTCH3 [30], [31]. Briefly, purified proteins were labeled with Alexa 700 succinimide. After removal of free label by gel filtration chromatography, the proteins were applied to ELISA plates coated overnight with target proteins (or BSA control) at 200 ng/ml in Tris-buffered saline (50 mM Tris, 150 mM NaCl) with 2 mM CaCl2 overnight at 4°C and blocked with 1% BSA in Tris-buffered saline with 2 mM CaCl_2_ for 1 h at room temperature. After an overnight incubation of labeled protein in TBS with 2 mM calcium and 0.05% Tween 20, plates were rapidly washed with the same buffer three times at room temperature and total probe bound to the plate was visualized using a LiCor flatbed infrared scanner. As a negative control, each experiment also tested binding of control Fc to proteins immobilized on the plate, and this background binding was subtracted from the signal of the test proteins. Results are presented by weight (as opposed to molarity) of proteins, since proteins may form multimers.

### Notch trans-endocytosis assay

This procedure was performed as previously described by Meng et al [31] with HRP-tagged NOTCH3 expressing 293 cells mixed with mouse fibroblast cells producing Jagged1 (stable L cell lines described above). The total NOTCH3 protein trans-endocytosed by mouse cells was quantified by western blotting of proteins after magnetic bead mediated cell depletion with the monoclonal antibody TRA1-85, which binds to human cells; this procedure produces cell populations that are quantitatively depleted of human cells and are exclusively mouse Jagged1 cells.

Endocytosis of HRP-tagged NOTCH3 protein was directly visualized by immunofluorescent staining of cocultures for HRP (to follow NOTCH3) and TRA1-85 (a human-specific marker to distinguish NOTCH3 producing 293 cells from mouse fibroblasts). TRA1-85 negative cells which expressed HRP-NOTCH3 were identified as cells that had trans-endocytosed protein from co-cultured human cells. An Olympus confocal microscope was used to capture images of cells with internalized HRP-NOTCH3.

### Statistical analysis

Results are displayed with standard deviations. All luciferase and quantitative PCR studies were done in groups of three. ANOVA testing was applied with statistically significant differences considered for p<0.05.

## Results

### vWF suffuses the vascular wall in small cerebral vessel disease

**Figure 1:**
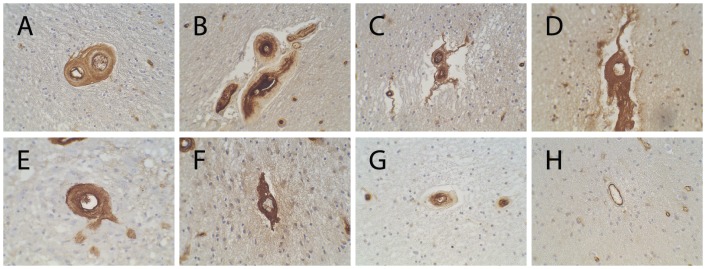
Arterial deposition of vWF in human cerebral small vessel disease. Pathologically thickened vessels of the cerebral white matter were examined by immunohistochemistry using antibodies against vWF in (A) vascular dementia with leukoencephalopathy and small vessel disease (73 year old man); (B) dementia and ischemic stroke (88 year old woman); (C) multiple infarct dementia (86 year old female); (D) sickle cell disease (25 year old female); (E) radiation necrosis following treatment for glioblastoma multiforme (63 year old female); (F) radiation necrosis following treatment for oligodendroglioma (36 year old man); (G) CADASIL (58 year old man); (H) control vessel with expected endothelial distribution of vWF. Photographs were taken at 400× magnification. Similar positive staining was seen with a mouse monoclonal antibody (Supplemental Figure 1). See Supplemental Figure 2 for additional control studies which feature (1) omission of primary antibody, (2) addition of vWF protein to block staining, and (3) verification of staining pattern with an independent polyclonal antibody.

In small cerebral arteries in CADASIL, vWF immunoreactivity is present not only in the endothelium but frequently expands into the entire thickened wall [24]. We found similar patterns of deposition of vWF in human cerebral small vessels in small vessel vascular dementia, multiple infarct dementia, sickle cell disease, and post-radiation vasculopathy (Figure 1). The transmural distribution of vWF protein in a wide range of cerebral SVD is similar to what we observed in CADASIL. The extension of vWF beyond the vascular endothelium was detected using both polyclonal (Figure 1) and monoclonal (Supplemental Figure 1) antibodies to mature vWF.

**Figure 2:**
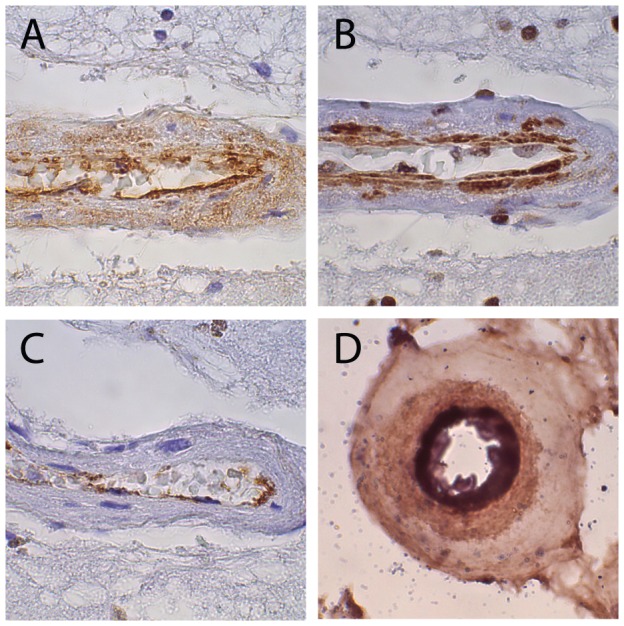
Mature vWF, smooth muscle actin, and vWF precursor protein expression in human small vessel disease. The same CADASIL cortical brain artery was examined by immunohistochemistry for mature vWF (A), smooth muscle actin (B), and vWF-pp (C). This vessel demonstrated transmural staining for mature vWF, subendothelial reactivity for SMA, and intimal-specific vWF-pp immunoreactivity. As in other SVD tissues, the staining for vWF-pp was not observed beyond the endothelium, but many segments of arteries were devoid of vWF-pp, suggesting inhomogeneity of endothelial coverage. (D) Confirmatory double stains were performed in to localize vWF-pp (dark blue stain on in inside of the artery) mature vWF (brown stain that extends into the arterial wall). Mature vWF is found in regions that lack vWF-pp staining. Photographs were taken at 1000×.

High powered images of small arteries in CADASIL show that vWF breaches the vascular endothelium and localizes to layers of the artery that express SMA (Figures 2A–B). To probe the origin of vWF production in arteries, we also stained for the vWF precursor peptide (vWF-pp), a peptide expressed in cells that synthesize vWF. Two patterns of vWF-pp expression emerged in brains with SVD. First, we found small arteries in which vWF-pp was not detected in the endothelium, though mature vWF was robustly expressed. Second, we found arteries where mature vWF penetrated the full thickness of the vessel but vWF-pp was restricted to the endothelium (Figures 2A and 2C). Double staining confirmed that vWF-pp was confined to the inner layer of vessels that expressed mature vWF which extended well beyond the inner cell layer (Figure 2D). The divergent distributions of mature vWF and vWF-pp suggest that ectopically deposited mature vWF in diseased tissue does not result from in situ synthesis.

### vWF blocks Notch signaling

In prior work, we found that vWF could both activate immediate early genes and downregulate genes important in maintenance of vascular smooth muscle maturity {Zhang, 2012 #133}. This suggested that vWF is capable of interacting with cell surface receptors to alter cell phenotype. Notch signaling plays an essential role in development of the vascular system and has been shown to modulate vascular smooth muscle gene expression. We therefore tested whether vWF could affect Notch signaling.

**Figure 3:**
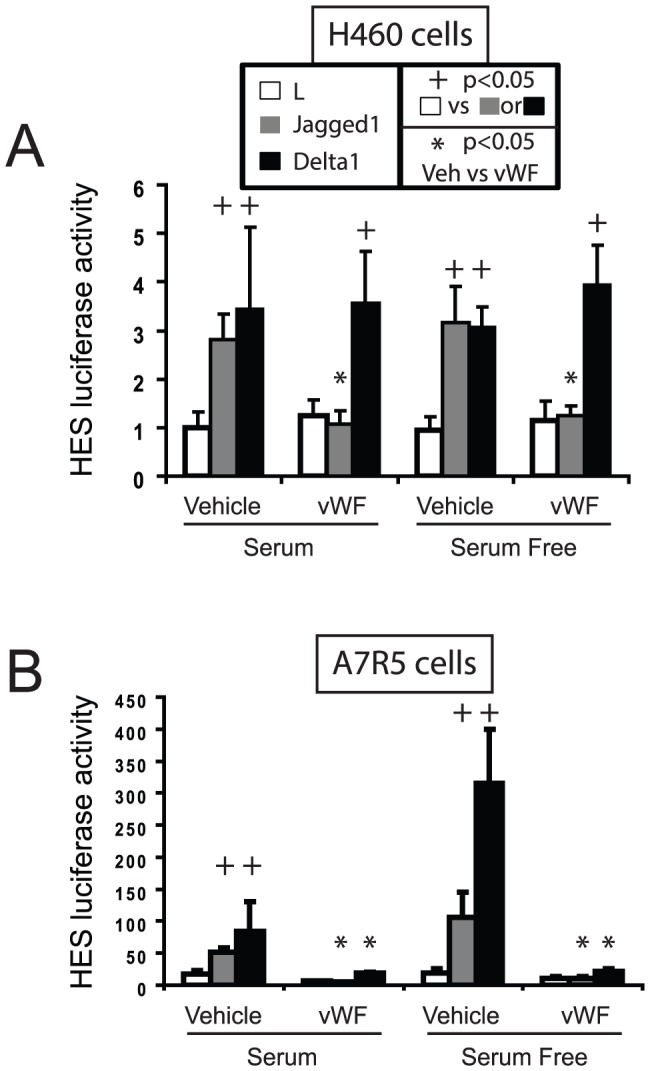
Effect of vWF on Notch signaling. A co-culture system was used to assess the effects of vWF on Notch signaling in H460 (A) and A7R5 (B) cells reflected by ligand stimulation of HES-luciferase. Experiments were performed in either serum-containing or serum-free media supplemented with vWF (200 ng/ml). Representative results from four experiments done in triplicate are shown. Please note significant changes (p<0.05) induced by Notch ligands (+) or by vWF (*).

We first tested the effect of vWF on NOTCH3 signaling using a co-culture assay described previously using H460 signal receiving cells (that only express the NOTCH3 isoform) and Notch ligand-expressing fibroblasts as signal sending cells [30], [31] [29]. As reported before, ligand expressing cells activated the HES-luciferase reporter. But in the presence of purified vWF, Jagged1-stimulated Notch signaling was markedly reduced (Figure 3A). Delta1-stimulated Notch signaling in H460 cells was not affected by vWF. The results were not affected by the presence of heat-inactivated bovine serum.

In a rat smooth muscle cell line (A7R5) (Figure 3B), Notch ligands stimulated HES-luciferase expression in A7R5 cells. A7R5 cells express all four Notch genes. Incubation of co-cultures with purified human vWF potently inhibited Notch signaling stimulated by either Jagged or Delta expressing fibroblasts. The results were similar in serum and serum-free conditions. These experiments demonstrated that vWF could inhibit Notch signaling in two independent cell lines.

### vWF reduces Notch-stimulated smooth muscle promoter activation

**Figure 4:**
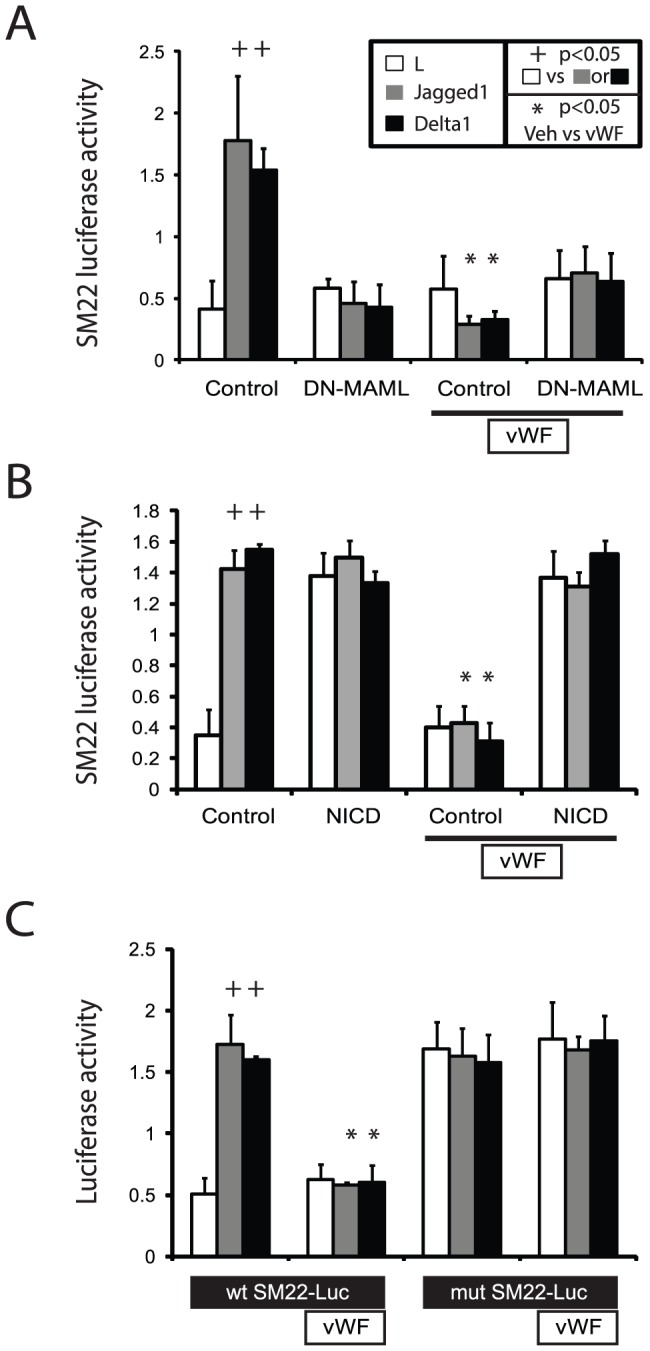
Effect of vWF on Notch-regulation of the SM22 promoter. The effect of vWF on a SM22-luciferase reporter was determined in experiments similar to those in Figure 3. The effect of DN-MAML was determined by cotransfection with reporters (A). The Notch3 NICD was used to activate Notch signaling by intracellular expression (B). vWF (200 ng/ml) was added to the media directly. (C) Notch-regulation of SM22-luciferase in which the CBF site at −396 was mutated (mutant SM22) was compared to the wild-type SM22 reporter (WT), with and without vWF. Significant changes (p<0.05) induced by Notch ligands (+) or by vWF (*) are marked. No differences were induced by Notch ligand in DN-MAML or NICD transfected groups, which were constitutively repressed and activated, respectively. No differences were induced in mutant SM22-luciferase expression by Notch ligands.

To determine whether vWF could inhibit smooth muscle transcription, we examined the protein's effect on cloned promoters from two well-characterized mature smooth muscle genes. A 0.4 kb SM22-luciferase reporter transfected into A7R5 cells [35] [36] was activated in our co-culture system (Figure 4A). Cotransfected DN-MAML peptide (a Notch-inhibitory peptide expressed as an EGFP fusion protein; [37]) completely blocked ligand-stimulated expression of the SM22 reporter. Addition of vWF to the media resulted in strong inhibition of Notch ligand-regulated SM22 reporter activity. However, vWF failed to further decrease promoter activity in DN-MAML-transfected cells.

Overexpressed Notch intracellular domain (NICD) from NOTCH3 [38] activated the SM22 promoter, and the activity of NICD of NOTCH3 was not affected by presentation of Notch ligands, which activate Notch through extracellular interactions (Figure 4B). Importantly, vWF failed to inhibit NICD activation of the SM22 promoter, suggesting that vWF selectively targets extracellular Notch activation.

Mutation of the CBF sequence (Notch responsive element) of the SM22 promoter (at −369 [28]) rendered the reporter constitutively active and unresponsive to Notch ligand stimulation (Figure 4C). Moreover, vWF failed to repress the mutant SM22 promoter (Figure 4C). These studies demonstrate that regions outside of the Notch-responsive CBF site of the SM22 promoter are not involved in downregulation of the SM22 promoter in the presence of vWF.

**Figure 5:**
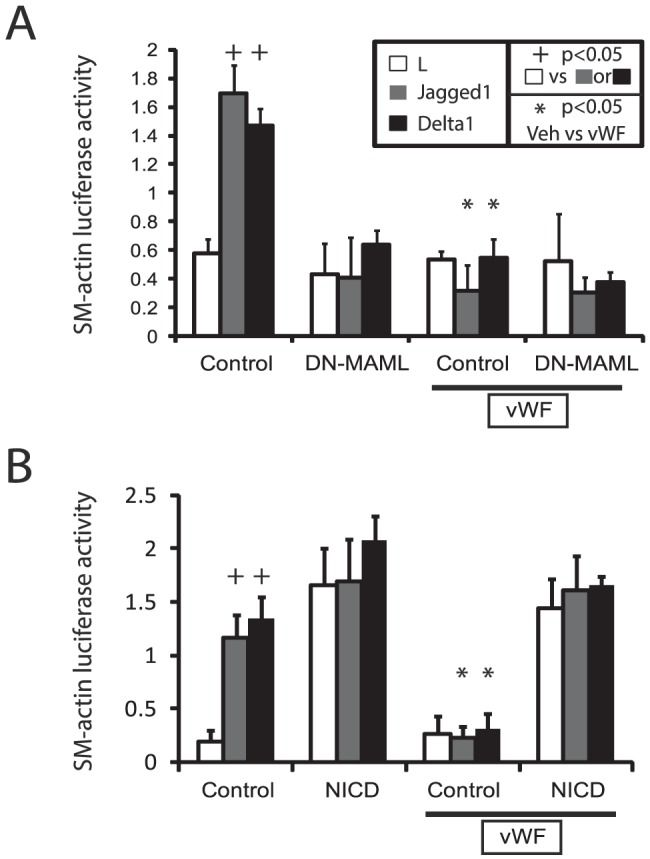
Effect of vWF on Notch-regulation of the SMA promoter. The effect of vWF on Notch regulation of a cloned SMA promoter driving firefly luciferase was determined as in Figure 4. vWF was added at 200 ng/ml. Significant changes (p<0.05) induced by Notch ligands (+) or by vWF (*) are marked. No differences were induced by Notch ligand in DN-MAML or NICD transfected groups, which were constitutively repressed and activated, respectively.

As reported [26], [27], the SMA promoter, investigated by using an SMA-luciferase reporter in this co-culture system, was induced by Notch ligands. vWF completely inhibited Notch activation of the SMA promoter. As in the case of the SM22 promoter, cell autonomous DN-MAML inhibition of Notch function was not affected by vWF (Fig 5A), and activation by transfection of NICD from NOTCH3 rendered the inhibitory functions of vWF ineffective (Fig 5B).

### vWF blocks Notch-dependent stimulation of smooth muscle marker expression

**Figure 6:**
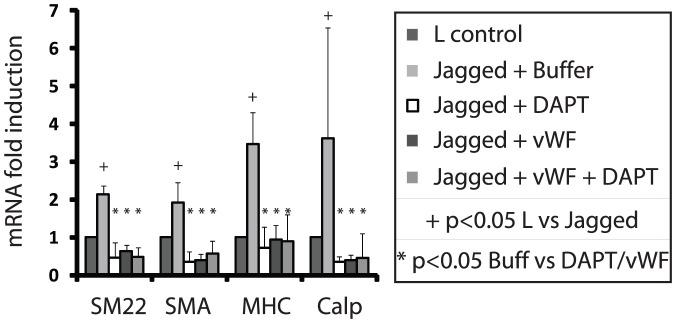
Smooth muscle gene regulation by Notch and vWF. The effects of Notch signaling and vWF on expression of four core smooth muscle genes in A7R5 cells were determined by quantitative reverse transcriptase PCR. Gamma-secretase inhibitor DAPT was applied or purified vWF (200 ng/ml) was added to the media. Significant changes (p<0.05) induced by Jagged are marked (+). DAPT or vWF incubation fully repressed Jagged stimulated gene expression (*; p<0.05).

**Figure 7:**
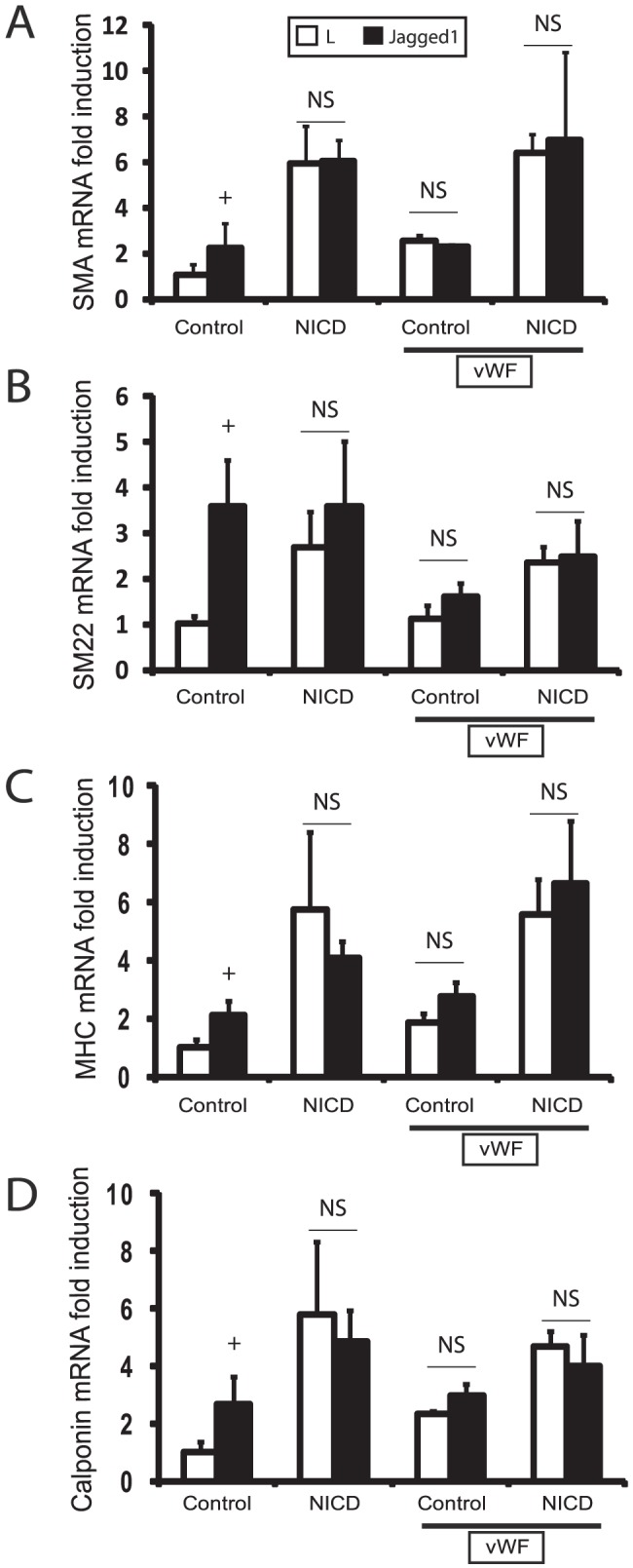
Effect of vWF on cell autonomous, intracellular Notch activation. A7R5 cells were first transfected with vector or NICD (from NOTCH3) prior to co-culture with ligand expressing cells (with or without vWF 200 ng/ml). Transcript quantitation was performed as in Figure 6 for: smooth muscle actin (A; SMA), SM22 (B), smooth muscle myosin heavy chain (C; MHC), and calponin (D). Significant changes (p<0.05) induced by Jagged1 are marked (+). In the presence of vWF, there was no upregulation of mRNA by Jagged1; in addition, after NICD transfection, transcripts were unaffected by Jagged1, with or without vWF (NS; no differences between L and Jagged). All transcript levels were greater in NICD transfected cells compared to control cells cocultured with L cells, with or without vWF (p<0.05; not marked).

Previous studies have shown that Notch activates smooth muscle differentiation markers [25] [26] [27] [28] [39]; accordingly, we detected upregulation of key mature smooth muscle transcripts encoding SM22, SMA, SM-MHC, and calponin in A7R5 cell lines cocultured with Jagged-expressing cells (Figure 6). DAPT inhibited the Jagged-stimulated upregulation of all four of these genes, and vWF prevented Notch stimulated activation of these transcripts to a degree that was similar to DAPT application (Figure 6). Conversely, endogenously expressed smooth muscle markers were activated by transfection of the NICD of NOTCH3. Activation of these genes by NICD bypassed the inhibitory effects of vWF (Figure 7). In summary, vWF is capable of inhibiting endogenous expression of four key smooth muscle genes (Figure 6–7); moreover, the inhibitory activity of vWF requires extracellular (non-cell autonomous) activation of Notch.

**Figure 8:**
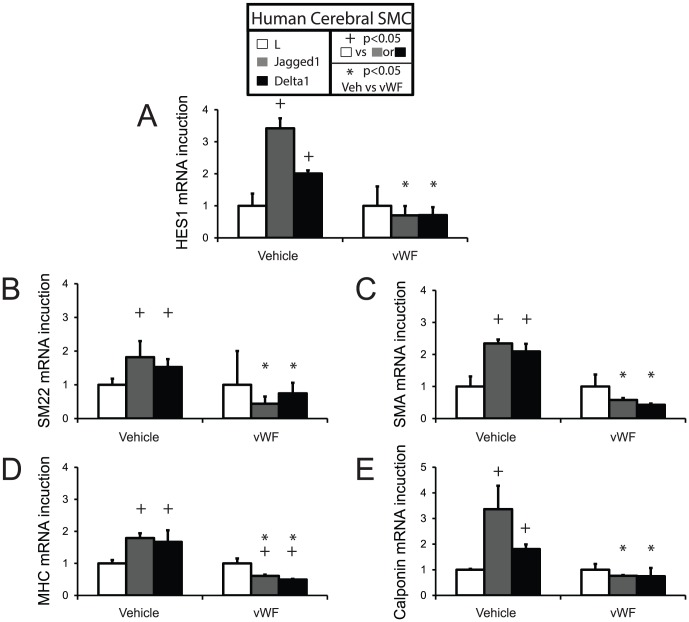
Effect of vWF on Notch-regulated gene expression in primary human brain vascular smooth muscle cells. Primary cultures of brain vascular smooth muscle cells were cocultured with Notch ligand producing cells (or control cells). In replicate groups, vWF (500 ng/ml) was added to cultures prior to analysis. RNA from three wells of cocultures was analyzed with human-specific primers using quantitative RT-PCR for expression of human HES1 (A), SM22 (B). SMA (C), MHC (D), and calponin (E); gene expression was normalized to cocultures with control L cells. Three replicates of the representative results displayed showed similar results. + indicates significant differences in expression between L (control) and Jagged or Delta stimulated cells (p<0.05). * indicates significant differences after incubation with vWF (p<0.05).

In addition, we tested the potential for vWF to block Notch-stimulated expression of smooth muscle genes in primary cultures of human cerebral vascular smooth muscle cells (Figure 8). Human primary cells stimulated with ligand-expressing mouse fibroblasts increased expression of the canonical Notch-responsive gene HES1 (Figure 8A). Similarly, smooth muscle markers SM22, SMA, MHC, and calponin were each activated by Notch ligands, demonstrating conservation of NOTCH signaling in human cerebral smooth muscle cells. Addition of vWF to co-cultures fully blocked the Notch-dependent activation of each of these markers.

### Interactions between vWF and Notch ectodomains

**Figure 9:**
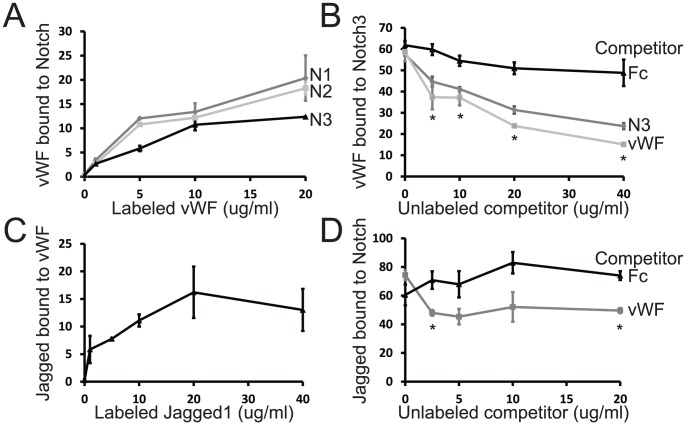
In vitro interactions between vWF and Notch/Jagged. (A) Wells coated with purified vWF (200 ng/ml concentration) were probed with Notch1–3 proteins fused to Fc. (B) Wells coated with vWF were incubated with labeled Notch3 in the presence of increasing concentrations of indicated proteins. (C) Wells coated with purified vWF were probed with purified rat Jagged-Fc. (D) To assess whether vWF could interfere with Notch3-Jagged interactions, we performed binding studies in the presence of unlabeled vWF or control Fc protein. Wells coated with unlabeled Jagged1-Fc were probed with labeled Notch3 in the presence of increasing concentrations of unlabeled vWF. The Y-axis corresponds to fluorescence units defined by the LiCor IR scanner used to detect dye-labeled protein probes after subtraction of signal generate by using an equivalent mass of Fc control protein. The molar concentrations of 10 ug/ml Notch1-3 and Jagged1 correspond to 125, 124, 140, and 71 nM, under the assumption they are in monomer form. Significant differences between Fc competition and vWF or Notch3 competition are denoted (* in (B, D), p<0.05).

Our previous studies have suggested that vWF interacts with extracellular components of smooth muscle cells [24]. In addition, since NICD expression bypasses vWF inhibition of Notch signaling, we reasoned that the ectodomain of Notch proteins may be targeted by vWF. To test whether Notch ectodomains could interact with vWF, we labeled purified vWF and applied the protein to plates coated with Notch ectodomain fragments. As shown in Figure 9A, vWF bound to Notch 1, 2, and 3; vWF did not bind to plates coated with Fc control proteins. The interaction between vWF and Notch3 was inhibited by coincubation with excess unlabeled vWF and Notch3-Fc protein (Figure 9B). In addition, Jagged1, but not control Fc protein, bound to vWF that was immobilized to plastic dishes (Figure 9C). To assess whether vWF interactions with extracellular Notch components could compete against Notch receptor-ligand binding, we tested the effect of unlabeled vWF on NOTCH3 binding to Jagged1. As shown in Figure 9D, vWF reduced the amount of Notch3-Jagged1 binding in vitro.

**Figure 10:**
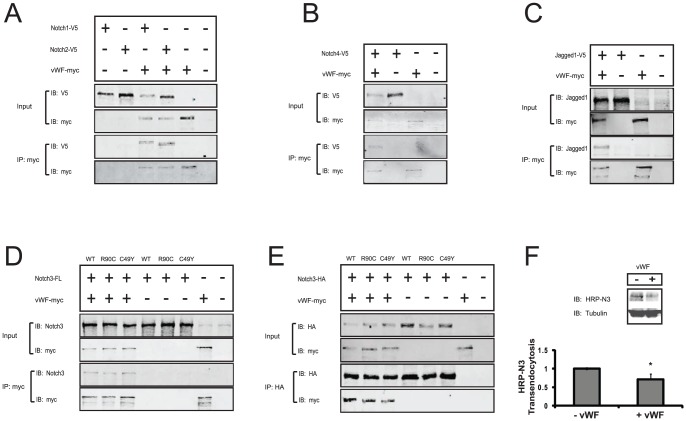
Interactions between vWF and Notch proteins in cultured cells. Co-immunoprecipitation assays were performed in transfected cultures to demonstrate molecular complex formation between vWF, Notch ectodomains, Jagged ectodomain, and full length NOTCH3 protein. (A–E) Cultured 293A cells were cotransfected with plasmids encoded vWF and/or Notch or Jagged1 ectodomains or full length NOTCH3, as indicated; vWF and ectodomain clones were C-terminally tagged to facilitate detection and pull down. Input cell lysates were immunoblotted (IB) to confirm expression. Monoclonal antibody-treated lysates were also immunoprecipitated with protein G-agarose. Precipitated proteins (IP) were analyzed by immunoblotting. Co-precipitation of vWF and CADASIL-causing NOTCH3 mutant proteins was assessed in (D–E). In negative control experiments (shown in each panel), IP of single transfections did not pull down proteins. Apparent molecular weights of proteins were >250 kDa (vWF; a doublet), approximately 180 kDa (Notch1-V5 and Notch2-V5), and 150 kDa (Notch3-HA and Notch4-V5). (F) vWF (200 ng/ml) was added to co-cultures of HRP- NOTCH3 and mouse fibroblasts to measure vWF-regulation of Notch3 transendocytosis. vWF reproducibly impaired trans-endocytosis of NOTCH3 into the signal-sending cells in three independent experiments (p<0.05).

Figure 10A–E shows results from co-immunoprecipitation studies employing overexpressed Notch ectodomains or Jagged and vWF. All four Notch ectodomains and Jagged coprecipitated with vWF. Full length NOTCH3 also formed complexes with vWF in cells. CADASIL mutations did not appreciably affect NOTCH3 binding to vWF, as measured by co-immunoprecipitation.

Previous studies of TSP2, which amplifies Notch signaling, demonstrate coupling between Notch activation and ectodomain transendocytosis [31]. In the case of TSP2, binding to NOTCH3 may enhance endocytosis of the receptor by an LRP1 dependent mechanism that amplifies Notch signaling. Because vWF strongly reduced Notch signaling and also bound to the ectodomain of Notch proteins, we suspected that this hemostatic protein could also inhibit Notch trans-endocytosis. Tagged NOTCH3 expressing cells were cocultured with ligand expressing fibroblasts. After immunodepletion of NOTCH3 producing cells, we quantitatively determined the amount of NOTCH3 internalized by ligand expressing cells. Incubation of cocultures with vWF significantly reduced transendocytosed NOTCH3 ectodomain (Figure 10F). Immunofluorescent staining of cocultured cells confirmed that vWF inhibited the uptake of Notch proteins into intracellular compartments of ligand producing cells (Supplemental Figure 3). These results are consistent with a model in which vWF inhibits NOTCH signaling via inhibition of extracellular NOTCH3 function, leading to reduction of Notch target gene expression.

## Discussion

Although vWF is best known as a hemostatic protein [40], when secreted into the deep layers of the vessel wall, it is unlikely to participate in hemostasis. Most recently, we have shown novel functions of vWF including activation of immediate early genes and suppression of smooth muscle marker genes [24]. A number of studies have raised the possibility that vWF may stimulate non-canonical activities that drive intimal thickening [41] [42] [43]. This report presents the new concept that vWF represses expression of genes encoding mature smooth muscle proteins by inhibiting Notch function.

Multiple lines of evidence presented herewith suggest that vWF targets Notch. Using cell co-culture to activate Notch signaling, we show that Notch increases levels of smooth muscle specific genes (in the absence of vWF). Previous results using either purified Notch ligand or overexpression of NICD have yielded conflicting data regarding Notch regulation of smooth muscle target genes [26], [44], [45], [46]. Importantly, our studies utilize a biologically relevant non-cell autonomous model to interrogate Notch signaling, which was also used by Doi et al [28] who arrived at similar results. We show, furthermore, that extracellular vWF inhibits Notch-mediated upregulation of these genes and their promoters. The inhibitory activity of vWF occurs at levels that are markedly lower than serum concentrations of vWF (50-fold lower), strongly suggesting that sub-serum levels of vWF present in the vessel wall could exert similar effects *in vivo*.

The repression of Notch signaling by vWF requires the Notch ectodomain, since gene activation by transfection of NICD of Notch3, which bypasses extracellular signaling, is not affected by vWF. Our demonstration that vWF interacts with all four of the ectodomains of Notch proteins provides additional evidence that vWF mediates Notch inhibition through an extracellular process. Finally, the ability of vWF to block Notch interactions with Jagged and to prevent trans-endocytosis of Notch3-ectodomain suggests that vWF interferes with Notch signaling by competitively inhibiting extracellular Notch receptor-ligand interactions.

Additional experiments are needed to characterize the full nature of Notch-vWF interactions. Each of the proteins used to study this interaction are in potentially multimerized states, and, therefore, our experiments have limited ability to determine precise affinity constants since the molarity of proteins are not precisely defined. An additional potential complication is that Notch-vWF interactions may be mediated by multiple domains of each protein. Further experiments using smaller, monomeric proteins could address affinity of complex formation and permit interaction domain mapping.

Previous work has implicated vWF in smooth muscle proliferation [41], [42], [43]. However, vWF impairment of Notch is probably not solely responsible for cell proliferative responses since chemical inhibition of Notch signaling with DAPT did not activate mitosis. A simple model for the function of vWF on smooth muscle cells posits that vWF modulates multiple signaling systems responsible separately for proliferation (presently unknown) and differentiation (eg. Notch). Certainly, the immense size and presence of multiple protein binding motifs within vWF raises the possibility that a large array of regulatory protein-protein interactions could take place between vWF and smooth muscle cell membrane receptors.

We observed highly consistent inhibition of Notch-regulated smooth muscle transcripts by vWF. However, a number of interesting findings were also noted that indicate a more complex situation. For example, we noted a modest increase in smooth muscle transcript levels in vector transfected cells treated with vWF in the absence of ligand (in the presence of L fibroblasts; Figure 7). Moreover, vWF exhibits unexpected Notch ligand-specific inhibitory effects in H460 cells (Figure 3A). We propose that the context and ligand-specific actions of vWF could be due to interactions with other, yet to be identified, surface proteins. Multiple differences between the three cell types (malignant human lung adenoma H460 cells, rat embryonic aortic smooth muscle line A7R5, and primary human brain vascular smooth muscle cells) used here make it difficult to unravel the mechanisms underlying ligand specific vWF action. Further work is needed to define additional signaling pathways of vWF. For example, vWF blockade may require additional membrane receptors or Notch modifications (eg. glycosylation) that confer inhibition of specific ligands; alternatively, vWF may differentially interact with ligand in a cell-specific manner.

Candidate targets that warrant experimental testing include integrins that may interact with both collagen and vWF [47]. In addition, the interaction between vWF and the TGF-beta signaling pathway that cooperates with Notch to regulate the contractile cell phenotype may be of significant interest [26]. Our work adds additional significance to recent studies that demonstrate that vWF/integrin partnering is a downstream effector of Notch-mediated smooth muscle investment of developing retinal arteries [48]. In that study by Scheppke et al, vWF was proposed to be an adhesion molecule for smooth muscle cells though interactions with integrins. Further work will be required to understand whether the observations made by Scheppke et al also involve vWF-mediated modulation of Notch signaling processes.

Our study grew out of initial observations that small vessels in CADASIL contain transmural deposits of vWF [24]. However, we have yet to discover unique interactions between mutant NOTCH3 and vWF. Both mutant and wildtype NOTCH3 proteins bind to vWF, and vWF alters Notch signaling in cells expressing wildtype receptors, suggesting that vWF effects on smooth muscle cells do not require NOTCH3 mutations. Rather, it is more likely that mutations in NOTCH3 result in secondary upregulation of vWF protein in CADASIL small arteries.

Since vWF can impair key smooth muscle genes through Notch inhibition, the effects of vWF in small vessels of the brain could be more generalized. A broad array of disorders of brain vessels have been associated with endothelial dysfunction, which results in increases in vWF secretion. We therefore posit that disease processes that affect the endothelium, which include those examined here (Figures 1 and 2), may cause increased deposition of vWF that ultimately permeates the entire vessel wall.

In a previous study, we suggested that vWF may be secreted from the endothelium into deeper layers of arteries, based on gradients of immunoreactivity that diminished in the media and adventitia [33]. To further characterize potential origins of vWF, we stained for vWF-pp, which is produced in cells that synthesize vWF. In SVD arteries with transmural mature vWF deposition, vWF-pp is confined to partially denuded endothelium without detectable subintimal or smooth muscle cells expression. This provides further support that vWF originates from either endothelial cells or the serum in small vessel disease.

A number of studies have shown a link between vWF and ischemic stroke [49], [50], [51], [52], and it has been presumed that excess vWF may increase thrombosis via a luminal mechanism. But, immunohistochemical studies shown here (Figures 1–2) demonstrate that vWF is found in significant quantities in the vessel wall in cerebral SVD of a variety of causes. In all of the brains that expressed transmural vWF, there was a notable depletion of cellularity of the thickened penetrating arteries including abnormal morphological distribution and potential migration of smooth muscle actin expressing cells (Figure 2), indicating a pathological process that results in significant remodeling and loss of smooth muscle cells. When considered together, our findings suggest that vWF may participate in loss of cells and deposition of matrix proteins in SVD via vWF-mediated inhibition of Notch signaling. Animal models of vWF deposition in brain vasculature will be essential to test this mechanism in vivo. If validated, mapping the Notch interacting domain of vWF and targeting this region could be considered as a strategy to intervene in cerebral SVD without altering bleeding risk.

## Supporting Information

Figure S1 **Immunohistochemical analysis of small vessel disease due to radiation necrosis.** Here we demonstrate patterns of vWF deposition in small cerebral arteries of a patient with radiation therapy for brain malignancy that are similar to those shown in Figure 2. The morphology of the vessel is highly abnormal, with little normal vascular architecture remaining. (A) vWF is highlighted using a monoclonal antibody, which showed transmural staining that was similar to results using polyclonal serum. (B) SMA was expressed in overlapping regions of the vessel in a serial section. It was distributed in clusters that were asymmetrically distributed in the vessel wall and that did not morphological resemble normal smooth muscle cells. Apparent ectopic appearance of SMA reactivity at the lining of the vessel is likely in due to endothelial loss (see Figure 2C). 1000× magnification.
(EPS)

Figure S2 **Control staining in brain samples with small vessel disease.** Brains from genetically proven CADASIL patients (A and C) and from a patient with radiation necrosis (B) were stained as in Figure 1, with the following modifications. A) Immunohistochemical procedures were performed without inclusion of primary antibody; this demonstrated no staining (1000× magnification). (B) We included 500 ng/ml of purified vWF with the primary antibody; this notably blocked the staining of vascular structures while increasing overall background (400× magnification). (C) An independent polyclonal vWF antibody gave similar staining profiles as in Figure 1 (400× magnification).
(EPS)

Figure S3 **Visualization of the effect of vWF on NOTCH3 transendocytosis.** Human 293 cell lines permanently transfected with HRP-NOTCH3 cDNA were cocultured with mouse L cells permanently transfected with Jagged1. Groups were treated with either vehicle (A–B, E–F) or 200 ng/ml human vWF (C–D, G–H). After 24 hours of coculture, cells were fixed and immunostained for HRP (rabbit polyclonal antibody shown in green), TRA1-85 (mouse monoclonal antibody shown in red), and DAPI (blue). Triple colored merged confocal images (A–D) and double color (red and blue; E–H) images of the same fields are shown for comparison. Double color panels, both at low (E, G) and high power (F, H) show two populations of cells (red cells and unlabeled cells). Human cells are TRA1-85 positive (red) and coexpress HRP-NOTCH3. Mouse cells making Jagged1 do not stain with TRA1-85 and are labeled only in blue. In vehicle treated cells, populations of Jagged1 expressing mouse cells display intracellular HRP-NOTCH3 immunoreactivity (green without red; A–B), consistent with trans-endocytosis of protein. Occasionally, TRA1-85 negative cells (white arrows) were observed that labeled very strongly for HRP-N3 (B). In cells exposed to vWF, we noted decreased HRP-N3 label in TRA1-85 negative cells (C–D). Strongly labeled cells were markedly decreased in vWF-treated cocultures (D).
(EPS)
